# Smoking, and to a lesser extent non-combustible nicotine use, is associated with higher levels of alcohol consumption and risky drinking

**DOI:** 10.1038/s41598-025-89750-2

**Published:** 2025-02-26

**Authors:** Sarah E. Jackson, Melissa Oldham, Claire Garnett, Jamie Brown, Lion Shahab, Sharon Cox

**Affiliations:** 1 Department of Behavioural Science and Health, University College London, 1-19 Torrington Place, London, WC1E 7HB UK; 2 SPECTRUM Consortium, Edinburgh, UK; 3 School of Psychological Science, University of Bristol, Bristol, UK

**Keywords:** Increasing and higher risk drinking, Alcohol, Tobacco, Smoking, Vaping, E-cigarettes, Heated tobacco, Nicotine pouches, Heat not burn, GP advice, Primary care, Quit attempts, Health care, Risk factors

## Abstract

This study aimed to estimate differences in alcohol consumption, receipt of alcohol brief intervention, and alcohol reduction attempts by smoking status and use of non-combustible nicotine (including e-cigarettes, nicotine replacement therapy, heated tobacco products, or nicotine pouches). Data were from a representative household survey of adults in England (*n* = 188,878). Participants who reported former or current smoking scored approximately 1 point higher, on average, on the AUDIT-C (which measures alcohol consumption) than those who had never regularly smoked (*B*_adj_=0.97 [95%CI 0.93–1.00] and 0.92 [0.87–0.96], respectively) and had double the odds of risky drinking (AUDIT-C ≥ 5: OR_adj_=2.04 [1.98–2.10] and 2.03 [1.97–2.10], respectively), while differences for those who did versus did not use non-combustible nicotine use were less pronounced (AUDIT-C: *B*_adj_=0.14 [0.08–0.21]; AUDIT-C ≥ 5: OR_adj_=1.09 [1.04–1.13]). Among participants who engaged in risky drinking, those who smoked (vs. not) were more likely to report receiving alcohol brief interventions, and those attempting to quit smoking (vs. not) were more likely to report alcohol reduction attempts. Overall, combustible and - less so - non-combustible nicotine use is associated with higher levels of alcohol consumption and risky drinking.

**Supplementary Information:**

The online version contains supplementary material available at 10.1038/s41598-025-89750-2.

## Introduction

Alcohol use is a leading risk factor for morbidity and mortality^1,2^. In England, one in three adults drinks at increasing and higher risk levels (defined as a score ≥ 5 on the Alcohol Use Disorders Identification Test—Consumption (AUDIT-C)^3^; hereafter referred to as ‘risky drinking’)^4^. Reducing alcohol consumption can reduce the risk of chronic diseases and increase healthy life expectancy^5^. UK clinical guidelines recommend that health professionals working in primary care screen patients for risky drinking and offer structured brief advice and/or referral to specialist treatment services^6^. Given the combined effects of alcohol and smoking on health harms^7–11^, understanding the extent to which people who smoke or use other forms of non-combustible nicotine drink more heavily, are less likely to make alcohol reduction attempts, or are less likely to receive advice on alcohol from their general practitioner (GP) is important for targeting brief intervention delivery.

Substantial evidence shows a strong correlation between alcohol and nicotine use. For example, in England, one in 20 adults (4.6%) both smoke and engage in risky drinking^12^. At the population level, time-series analyses show changes in the prevalence of tobacco smoking are positively associated with changes in the prevalence of risky drinking^13^. At the individual level, recent studies in England have documented reciprocal associations between smoking and risky drinking^12,14^. Smoking prevalence is much higher among those who drink more heavily: 57.9% among adults at risk of alcohol dependence compared with 18.7% among those who drink but are not at risk of dependence and 19.2% among those who do not drink at all^14^. In addition, among people who engage in risky drinking, those who smoke have significantly higher AUDIT scores than those who do not (12.8 vs. 10.9)^12^. It is not clear how far patterns of alcohol consumption differ between people who have quit smoking and those who have never regularly smoked, or how they differ by level of dependence among people who currently smoke. These differences are important to understand, given alcohol use is a common trigger of relapse to smoking^15^, and the health risks associated with both alcohol and smoking are greater among those who are more dependent^16,17^.

Much of the research on the association between alcohol and nicotine has focused on tobacco use – specifically cigarette smoking^18^. Less is known about co-use of alcohol and non-combustible nicotine products (i.e. e-cigarettes, nicotine replacement therapy (NRT), heated tobacco products, and nicotine pouches), which represent a substantial and growing portion of nicotine use^19^. Pharmacological actions common to both alcohol and nicotine likely play a role in dual use^20,21^. Both substances act on the mesolimbic dopamine system (the brain’s reward system), creating a rewarding and reinforcing effect^20^. In addition, neuroadaptation occurs with chronic use of either substance, leading to cross-tolerance, where tolerance to one substance influences tolerance to the other – meaning use of either drug enhances the reinforcing properties of the other^20^. Shared genetic factors also appear to contribute to co-occurrence, as certain genes may predispose people to a heightened susceptibility for both alcohol and nicotine dependence^22^. Examining how patterns of alcohol use differ according to use of non-combustible nicotine products can offer insights into potential consequences of the evolving nicotine market on alcohol consumption.

Alcohol and tobacco each have their own associated risks to health, and while non-combustible nicotine products tend to be substantially less harmful than combustible tobacco they are not entirely without risk^23^. Combined use of alcohol and nicotine (especially in the form of combustible tobacco) can have additive or multiplicative effects on health risks^7–11^, making people who co-use an important target for brief intervention. However, evidence suggests health professionals seem to target smoking more often than drinking^24,25^, in part because alcohol is perceived as less harmful than smoking, a more sensitive issue to raise with patients, and intervention is perceived as being less effective^26^. A recent study found that most adults who engage in risky drinking have not received an alcohol brief intervention in the past year, but this proportion was higher among those who smoked than those who did not (8.7% vs. 6.4%),^12^ suggesting health professionals may be more likely to offer advice on alcohol when patients also smoke. However, this difference may have been attributable to differences in sociodemographic characteristics or level of alcohol consumption, which requires further exploration.

Regardless of whether they receive a brief intervention, many people make attempts to cut down on their drinking or stop smoking each year^4,27^. A recent study suggested the proportion making a serious attempt to restrict their drinking the past year was lower among adults who engage in risky drinking and currently smoke than among adults who engage in risky drinking who do not smoke (13.0% vs. 17.1%).^12^ Again, it is possible that this difference could be explained by differences in the characteristics of those who do and do not smoke. We also do not know whether rates of alcohol reduction attempts vary among people who smoke according to their level of tobacco dependence, which is an important consideration given the dose-dependent health risks of both drinking and smoking^16,17^. Neither is it clear whether people who co-use alcohol and tobacco who attempt to quit smoking are more or less likely to also make an alcohol reduction attempt.

In summary, it is currently unclear whether adults who engage in risky drinking and smoke tobacco have different patterns of alcohol consumption, or are more or less likely to make a reduction attempt or receive a brief intervention, than those who engage in risky drinking but have never smoked or have quit smoking. It is also not known whether similar associations exist for use vs. non-use of non-combustible nicotine products, such as e-cigarettes. Using data from a nationally representative survey of adults in England, this study aimed to estimate differences in alcohol consumption, receipt of an alcohol brief intervention, and alcohol reduction attempts by smoking status and use of non-combustible nicotine. Secondary aims were to explore differences in alcohol consumption and alcohol reduction attempts among people who currently smoke by level of tobacco dependence, and differences in alcohol reduction attempts by whether they made an attempt to quit smoking.

## Results

A total of 192,313 adults (≥ 18 years) responded to the Smoking and Alcohol Toolkit Study between March 2014 and December 2023. We excluded 3,435 (1.8%) with missing data on alcohol consumption or smoking status (2833 were missing alcohol consumption, 476 smoking status, and 126 both alcohol consumption and smoking status), leaving a final sample for analysis of 188,878 participants. The sample had a weighted mean age of 48.0 years (range 18–99 years), 50.9% were women, and 44.4% were from less advantaged social grades (C2DE). Table S1 provides a summary of the sample characteristics and missing data on each variable. Table 1 shows sample characteristics by smoking status and use of non-combustible nicotine.

**Table 1:** Weighted sample characteristics by smoking status and use of non-combustible nicotine.

	Smoking status			Use of non-combustible nicotine	
	Never	Former	Current	No	Yes
Unweighted *N*	116,553	40,595	31,730	173,125	15,753
Age					
Mean (SD)	47.2 (18.7)	55.0 (17.5)	42.1 (16.5)	48.5 (18.7)	42.1 (15.7)
18–24	13.9 [13.7–14.1]	4.1 [3.9–4.4]	16.5 [16.1–17.0]	12.0 [11.9–12.2]	15.2 [14.6–15.8]
25–34	17.2 [17.0–17.5]	11.5 [11.1–11.8]	23.8 [23.3–24.3]	16.5 [16.3–16.7]	23.5 [22.8–24.3]
35–44	16.4 [16.1–16.6]	14.9 [14.6–15.4]	18.0 [17.5–18.5]	16.1 [15.9–16.3]	19.4 [18.7–20.1]
45–54	17.0 [16.8–17.3]	17.8 [17.4–18.2]	17.6 [17.1–18.0]	17.1 [16.9–17.3]	18.9 [18.2–19.6]
55–64	14.3 [14.1–14.5]	17.4 [17.0–17.7]	12.6 [12.2–13.0]	14.8 [14.6–15.0]	13.1 [12.6–13.6]
≥65	21.1 [20.9–21.4]	34.3 [33.9–34.8]	11.5 [11.2–11.8]	23.4 [23.2–23.6]	9.9 [9.5–10.4]
Gender					
Men	46.5 [46.1–46.8]	52.3 [51.7–52.8]	53.3 [52.7–53.8]	48.5 [48.2–48.7]	53.0 [52.2–53.9]
Women	53.3 [53.0–53.6]	47.5 [47.0–48.1]	46.3 [45.7–46.9]	51.3 [51.0–51.6]	46.4 [45.6–47.2]
Other	0.3 [0.2–0.3]	0.2 [0.2–0.3]	0.4 [0.4–0.5]	0.2 [0.2–0.3]	0.6 [0.5–0.7]
Occupational social grade					
ABC1 (more advantaged)	59.7 [59.4–60.0]	56.6 [56.1–57.1]	39.9 [39.3–40.5]	56.5 [56.3–56.8]	46.0 [45.1–46.8]
C2DE (less advantaged)	40.3 [40.0–40.6]	43.4 [42.9–43.9]	60.1 [59.5–60.7]	43.5 [43.2–43.7]	54.0 [53.2–54.9]
Region					
North	27.0 [26.8–27.3]	29.0 [28.5–29.5]	30.6 [30.1–31.1]	27.6 [27.4–27.8]	33.0 [32.2–33.7]
Midlands	30.7 [30.5–31.0]	29.1 [28.7–29.6]	29.9 [29.3–30.4]	30.4 [30.1–30.6]	29.0 [28.2–29.7]
South	42.2 [41.9–42.5]	41.9 [41.3–42.4]	39.5 [38.9–40.1]	42.0 [41.8–42.3]	38.1 [37.3–38.9]
Smoking status					
Never	–	–	–	66.9 [66.7–67.2]	6.9 [6.5–7.3]
Former	–	–	–	19.5 [19.3–19.7]	36.0 [35.2–36.8]
Current	–	–	–	13.5 [13.3–13.7]	57.1 [56.3–57.9]
Use of non-combustible nicotine					
No	99.0 [99.0–99.1]	85.0 [84.6–85.4]	71.2 [70.6–71.7]	–	–
Yes	1.0 [0.9–1.0]	15.0 [14.6–15.4]	28.8 [28.3–29.4]	–	–

Data are shown as weighted percentages with 95% confidence intervals, unless otherwise specified. There were some missing data on gender (Table S1); valid percentages are shown for ease of interpretation.

### Alcohol consumption

We analysed differences in alcohol consumption among all adults in the sample.

#### Differences by smoking status

Adults who reported current or former smoking had higher mean AUDIT-C scores than those who had never regularly smoked and a higher proportion engaged in risky drinking (Table 2). After adjustment for sociodemographic characteristics, survey year, and use of non-combustible nicotine, mean AUDIT-C scores were approximately 1 point higher and the odds of risky drinking were twice as high among those reporting current or former smoking than never smoking (Table 2). The pattern of results was similar when we excluded participants who did not drink at all (Table S2).

#### Differences by non-combustible nicotine use

Adults who used non-combustible nicotine (i.e., e-cigarettes, NRT, heated tobacco products, or nicotine pouches) had higher mean AUDIT-C scores and prevalence of risky drinking than those who did not (Table 2). Differences were smaller than those observed by smoking status and were largely, but not entirely, explained by covariates (specifically, smoking status; 57.1% of those who used non-combustible nicotine reported current smoking and 36.0% former smoking, compared with 13.5% and 19.5% who did not; Table 1). After adjustment for sociodemographic characteristics and survey year, the mean AUDIT-C score was 0.7 points higher and the odds of risky drinking were two-thirds higher among those who did vs. did not use non-combustible nicotine (Table 2 footnote). After additional adjustment for smoking status, the mean AUDIT-C score was 0.14 points higher and the odds of risky drinking were 9% higher (Table 2). The pattern was similar when we excluded participants who did not drink at all (Table S2).

#### Differences by level of tobacco dependence

Among adults who currently smoked, mean AUDIT-C scores and the proportion who engaged in risky drinking were similar among those who rated their urges to smoke over the past 24 h between ‘not at all’ and ‘very strong’ but were higher among those reporting ‘extremely strong’ urges to smoke (Table 2). After adjustment for sociodemographic characteristics and survey year, the mean AUDIT-C score was half a point higher among those with the strongest vs. weakest urges to smoke and the odds of risky drinking were a third higher (Table 2). The pattern was similar when we excluded participants who did not drink at all (Table S2).

**Table 2:** Alcohol consumption among adults in England, by smoking status, use of non-combustible nicotine, and (among those who reported current smoking) strength of urges to smoke.

	*N* ^a^	AUDIT-C score			Risky drinking (AUDIT-C ≥ 5)		
		Mean [95%CI]	B [95%CI]	B_adj_ [95%CI]^b^	% [95%CI]	OR [95%CI]	OR_adj_ [95%CI]^b^
All adults							
Smoking status							
Never	116,553	2.73 [2.71–2.74]	Ref	Ref	23.8 [23.5–24.0]	Ref	Ref
Former	40,595	3.70 [3.66–3.73]	0.97 [0.93; 1.00]	0.97 [0.93; 1.00]^c^	36.3 [35.8–36.8]	1.83 [1.78; 1.88]	2.04 [1.98; 2.10]^c^
Current	31,730	3.65 [3.61–3.69]	0.92 [0.88; 0.96]	0.92 [0.87; 0.96]^c^	38.6 [38.0–39.2]	2.02 [1.96; 2.07]	2.03 [1.97; 2.10]^c^
Use of non-combustible nicotine							
No	173,125	3.02 [3.00–3.03]	Ref	Ref	27.9 [27.6–28.1]	Ref	Ref
Yes	15,753	3.82 [3.76–3.88]	0.80 [0.74; 0.86]	0.14 [0.08; 0.21]^d^	40.6 [39.7–41.4]	1.77 [1.71; 1.83]	1.09 [1.04; 1.13]^d^
Adults who currently smoked							
Strength of urges to smoke							
Not at all	4,628	3.95 [3.85–4.06]	Ref	Ref	42.8 [41.3–44.4]	Ref	Ref
Slight	5,619	3.69 [3.59–3.78]	-0.22 [-0.35; -0.10]	-0.08 [-0.20; 0.04]	39.8 [38.4–41.1]	0.90 [0.83; 0.97]	0.97 [0.90; 1.05]
Moderate	13,471	3.51 [3.45–3.57]	-0.39 [-0.50; -0.29]	-0.09 [-0.19; 0.01]	36.9 [36.0–37.8]	0.81 [0.76; 0.86]	0.96 [0.89; 1.02]
Strong	5,146	3.60 [3.49–3.70]	-0.34 [-0.47; -0.20]	0.00 [-0.13; 0.13]	37.7 [36.3–39.2]	0.82 [0.76; 0.89]	1.00 [0.92; 1.09]
Very strong	1,559	3.70 [3.50–3.90]	-0.22 [-0.44; 0.00]	0.09 [-0.12; 0.30]	37.8 [35.1–40.4]	0.82 [0.73; 0.93]	0.98 [0.86; 1.12]
Extremely strong	739	4.28 [3.95–4.60]	0.33 [0.00; 0.66]	0.53 [0.20; 0.86]	45.3 [41.4–49.2]	1.11 [0.94; 1.30]	1.31 [1.09; 1.56]

CI, confidence interval. OR, odds ratio.

^a^Unweighted sample size.

^b^All models were adjusted for age, gender, occupational social grade, and survey year (modelled non-linearly using restricted cubic splines, three knots). Models with smoking status as the exposure were also adjusted for use of non-combustible nicotine. Models with use of non-combustible nicotine as the exposure were also adjusted for smoking status.

^c^ Without adjustment for use of non-combustible nicotine: former smoking *B*_adj_ 0.99 [95%CI 0.95; 1.02] and OR_adj_ 2.06 [95%CI 2.00; 2.12]; current smoking *B*_adj_ 0.95 [95%CI 0.91; 1.00] and OR_adj_ 2.08 [95%CI 2.02; 2.14].

^d^ Without adjustment for smoking status: *B*_adj_ 0.70 [95%CI 0.64; 0.76] and OR_adj_ 1.64 [95%CI 1.57; 1.70].

### Receipt of alcohol brief intervention

We analysed differences in receipt of alcohol brief intervention among adults who engaged in risky drinking and visited their GP surgery in the past year.

#### Differences by smoking status

The proportion who recalled receiving any advice on alcohol – while overall very low – was highest among those who currently smoked and lowest among those who had never regularly smoked (Table 3). Descriptive data on the types of advice received are shown in Table S3. After adjustment for sociodemographic characteristics, survey year, and level of alcohol consumption, those who currently smoked had 71% higher odds of reporting alcohol brief intervention than those who had never regularly smoked, but odds were similar among those reporting former and never smoking (Table 3).

#### Differences by non-combustible nicotine use

The proportion who recalled receiving any advice on alcohol was also higher among those who used non-combustible nicotine than those who did not (Table 3). After adjustment for sociodemographic characteristics, survey year, and level of alcohol consumption, those who used non-combustible nicotine had 30% higher odds of reporting alcohol brief intervention than those who did not (Table 3 footnote), but this association was attenuated and not statistically significant after adjustment for smoking status (Table 3).

**Table 3:** Receipt of an alcohol brief intervention among adults in England who engaged in risky drinking and visited their GP, by smoking status and use of non-combustible nicotine.

	*N* ^a^	Received alcohol brief intervention^b^		
		% [95%CI]	OR [95%CI]	OR_adj_ [95%CI]^c^
Adults who engaged in risky drinking (AUDIT-C ≥ 5) who visited their GP				
Smoking status				
Never	15,776	2.04 [1.81–2.28]	Ref	Ref
Former	9,382	2.92 [2.55–3.29]	1.44 [1.21; 1.72]	1.10 [0.91; 1.32]^d^
Current	6,657	4.73 [4.20–5.27]	2.38 [2.02; 2.81]	1.71 [1.42; 2.07]^d^
Use of non-combustible nicotine				
No	28,276	2.70 [2.50–2.89]	Ref	Ref
Yes	3,539	4.22 [3.51–4.94]	1.59 [1.32; 1.93]	1.09 [0.88; 1.35]^e^

CI, confidence interval. OR, odds ratio.

^a^ Unweighted sample size.

^b^ Received any advice on alcohol. Descriptive data on the types of advice received are shown in Table S3.

^c^ All models were adjusted for age, gender, occupational social grade, survey year (modelled non-linearly using restricted cubic splines, three knots), and level of alcohol consumption (AUDIT-C score). The model with smoking status as the exposure was also adjusted for use of non-combustible nicotine. The model with use of non-combustible nicotine as the exposure was also adjusted for smoking status.

^d^ Without adjustment for use of non-combustible nicotine: former smoking OR_adj_ 1.11 [95%CI 0.93; 1.33]; current smoking OR_adj_ 1.75 [95%CI 1.47; 2.09].

^e^ Without adjustment for smoking status: OR_adj_ 1.30 [95%CI 1.06; 1.59].

### Alcohol reduction attempts

We analysed differences in alcohol reduction attempts among adults who engaged in risky drinking.

#### Differences by smoking status

The proportion who reported making ≥ 1 alcohol reduction attempt in the past year was highest among those who reported former smoking and lowest among those who currently smoked (Table 4). After adjustment for sociodemographic characteristics, survey year, and level of alcohol consumption, former smoking was associated with 14% higher odds of making ≥ 1 alcohol reduction attempt and current smoking was associated with 26% lower odds, relative to never smoking (Table 4).

#### Differences by non-combustible nicotine use

The proportion who reported making ≥ 1 alcohol reduction attempt was slightly higher among those who used non-combustible nicotine than those who did not (Table 4). After adjustment for sociodemographic characteristics, survey year, level of alcohol consumption, and smoking status, the odds of making ≥ 1 alcohol reduction attempt were 13% higher among those who used non-combustible nicotine than those who did not (OR_adj_=1.13 [95%CI 1.05; 1.22]) (Table 4).

#### Differences by level of tobacco dependence

Among those who currently smoked, the proportion who reported making ≥ 1 alcohol reduction attempt varied by strength of urges to smoke in the past 24 h (Table 4). It was lowest among those who reported moderate urges and highest among those who reported no urges or extremely strong urges. After adjustment for sociodemographic characteristics, survey year, and level of alcohol consumption, the odds of making ≥ 1 alcohol reduction attempt were 22% lower among those who reported moderate urges than those who reported no urges to smoke; other groups did not differ significantly from those who reported no urges (Table 4).

#### Differences by attempts to quit smoking

Among those who smoked in the past year, the proportion who reported making ≥ 1 alcohol reduction attempt in the past year was higher among those who reported making ≥ 1 attempt to quit smoking over this period (Table 4). After adjustment for sociodemographic characteristics, survey year, and level of alcohol consumption, those who tried to quit smoking had 46% higher odds of also trying to reduce their alcohol consumption than those who did not try to quit smoking (Table 4).

**Table 4:** Past-year alcohol reduction attempts among adults in England who engaged in risky drinking, by smoking status, use of non-combustible nicotine, and (among those who currently smoked) strength of urges to smoke and past-year attempts to quit smoking.

	*N* ^a^	Made ≥ 1 alcohol reduction attempt in the past year		
		% [95%CI]	OR [95%CI]	OR_adj_ [95%CI]^b^
Adults who engaged in risky drinking (AUDIT-C ≥ 5)				
Smoking status				
Never	27,178	28.2 [27.6–28.8]	Ref	Ref
Former	14,512	33.8 [33.0–34.7]	1.30 [1.24; 1.37]	1.14 [1.08; 1.21]^c^
Current	12,020	24.4 [23.5–25.3]	0.82 [0.78; 0.87]	0.74 [0.70; 0.79]^c^
Use of non-combustible nicotine				
No	47,377	28.5 [28.0–28.9]	Ref	Ref
Yes	6,333	30.9 [29.6–32.3]	1.12 [1.05; 1.02]	1.13 [1.05; 1.22]^d^
Adults who engaged in risky drinking and currently smoked				
Strength of urges to smoke				
Not at all	1,958	27.4 [25.1–29.6]	Ref	Ref
Slight	2,192	24.7 [22.7–26.8]	0.87 [0.75; 1.00]	0.89 [0.77; 1.03]
Moderate	4,852	22.4 [21.0–23.7]	0.72 [0.64; 0.82]	0.78 [0.69; 0.88]
Strong	1,912	26.0 [23.8–28.3]	0.89 [0.77; 1.04]	0.95 [0.81; 1.11]
Very strong	591	24.6 [20.5–28.7]	0.87 [0.69; 1.10]	0.86 [0.68; 1.10]
Extremely strong	325	27.5 [21.7–33.4]	0.93 [0.69; 1.26]	0.80 [0.57; 1.12]
Adults who engaged in risky drinking and smoked in the past year				
Made ≥ 1 attempt to quit smoking in the past year				
No	8,702	22.8 [21.8–23.8]	Ref	Ref
Yes	4,168	30.3 [28.7–31.9]	1.47 [1.34; 1.62]	1.46 [1.32; 1.61]

CI, confidence interval. OR, odds ratio.

^a^ Unweighted sample size.

^b^ All models were adjusted for age, gender, occupational social grade, survey year (modelled non-linearly using restricted cubic splines, three knots), and level of alcohol consumption (AUDIT-C score). The model with smoking status as the exposure was also adjusted for use of non-combustible nicotine. The model with use of non-combustible nicotine as the exposure was also adjusted for smoking status.

^c^ Without adjustment for use of non-combustible nicotine: former smoking OR_adj_ 1.17 [95%CI 1.11; 1.23]; current smoking OR_adj_ 0.77 [95%CI 0.72; 0.82].

^d^ Without adjustment for smoking status: OR_adj_ 1.05 [95%CI 0.98; 1.13].

## Discussion

Relative to adults who had never regularly smoked, those who reported former or current smoking (particularly those who reported the highest levels of dependence) reported greater mean alcohol consumption and had higher odds of risky drinking. To a lesser extent, adults who used non-combustible nicotine also reported higher mean alcohol consumption than those who did not and higher odds of risky drinking and making ≥ 1 past-year alcohol reduction attempt. Among adults who engaged in risky drinking, those who currently smoked were more likely than those who had never regularly smoked to report receipt of alcohol brief intervention and those who reported former smoking were more likely to report making ≥ 1 past-year alcohol reduction attempt. Among adults who engaged in risky drinking and currently smoked, those who attempted to quit smoking had higher odds of also attempting to reduce their alcohol consumption than those who did not. This study adds to and extends the evidence base on alcohol and nicotine use, with three key findings.

### Alcohol consumption

The first key finding is that smoking is much more strongly associated with alcohol consumption than use of non-combustible nicotine is. Participants who reported former or current smoking scored approximately 1 point higher, on average, on the AUDIT-C than those who had never regularly smoked, and had double the odds of risky drinking. By comparison, those who used non-combustible nicotine scored approximately 0.1 points higher on the AUDIT-C than those who did not (a small difference) and had 9% higher odds of risky drinking.

The reasons for this pattern of findings are not clear. It could be linked to non-combustible nicotine use being a newer behaviour compared with smoking. The close cultural relationship between smoking and drinking in the UK has developed, and been fostered by marketing, over decades^28^. In contrast, the majority of non-combustible products are relatively new to the market and have only been available during a period with tighter marketing regulations. Relatedly, the relationship between smoking and drinking develops within many individuals over a lifetime of consumption. The majority of people who use non-combustible nicotine will have a much shorter history of use (given how long the products have been on the market) than the average person who smokes.

Another possible explanation may lie in the different risk profile of non-combustible nicotine products relative to combustible tobacco. Non-combustible nicotine is less harmful than smoking^23,29^–it is possible that people who opt for harm reduction in nicotine also try to reduce their harm from drinking. In addition, people who try to quit smoking and transition to non-combustible nicotine products may be less likely to be successful if they continue to drink heavily, as alcohol lowers people’s inhibitions and can trigger relapse to smoking^15^.

Finally, although all the analyses included adjustment for sociodemographic characteristics, it is possible that smoking is a stronger indicator of the types of people who are more likely to drink more, and unmeasured confounding (e.g., by variables such as propensity for risk-taking behaviour) persists in our analyses.

### Alcohol brief intervention

The second key finding is that adults who engage in risky drinking are more likely to report receiving alcohol brief intervention in primary care if they smoke than if they do not. While this pattern has been observed previously^12^, our results show it persists after adjustment for sociodemographic characteristics and level of alcohol consumption and other nicotine use. The difference was substantial: of adults who engaged in risky drinking, those who smoked had 71% higher odds of reporting receiving an alcohol brief intervention when they visited their GP than those who had never regularly smoked.

There are several possible explanations. Adults drinking at risky levels who smoke are much more likely to have comorbid health conditions^7^, which may prompt delivery of alcohol brief intervention or result in more frequent primary care attendance, offering greater opportunity to discuss their alcohol use. As health professionals more commonly deliver brief intervention on smoking than alcohol^24,25^, the higher receipt of alcohol brief intervention among people who smoke may also reflect health professionals mentioning alcohol consumption in the context of trying to stop smoking or being likely to mention alcohol if patients are receptive to advice on smoking. Interestingly, a recent paper suggested that while patients who both smoke and engage in risky drinking are more likely to report receiving an alcohol brief intervention than those who do not smoke, they are *less* likely to receive a smoking brief intervention than those who do not drink riskily^12^. This has important implications for smoking cessation: it is imperative that alcohol brief interventions are delivered alongside, not instead of, smoking brief interventions.

### Attempts to reduce alcohol consumption

The final key finding is that attempts to stop smoking are associated with attempts to reduce alcohol consumption. Among participants who had smoked in the past year, those who tried to quit smoking had 46% higher odds of reporting an alcohol reduction attempt than those who did not.

This is in keeping with the observation that participants who currently smoked were more likely to receive advice to cut down on alcohol consumption. Alcohol intake is a common trigger of relapse to smoking^15^ and people who smoke are commonly advised to avoid alcohol during a quit attempt, which may prompt those who try to stop smoking to also reduce their alcohol consumption to support their smoking cessation goal. A previous study suggested attempts to stop smoking and to reduce drinking happen concurrently: it found people who reported starting a quit attempt in the last week reported lower alcohol consumption, including less frequent heavy episodic drinking, and were more likely to report currently attempting to reduce their alcohol consumption than people who smoked but had not tried to stop in the last week^30^.

Our results also showed that participants who reported former smoking were more likely than those who currently or had never regularly smoked to report trying to cut back on their drinking. This finding may have been driven by self-selection if reducing alcohol consumption increases the chances that an attempt to stop smoking is successful (i.e., those who attempt to stop smoking who cut down drinking may be more likely to succeed, while those who do not cut down drinking are more likely to continue smoking).

### Strengths and limitations

Strengths of this study include the large, nationally representative sample and breadth of data on nicotine and alcohol use. There were also limitations.

All data were self-reported and measures of alcohol brief intervention and attempts to quit smoking and reduce alcohol consumption relied on recall of the past 12 months, introducing scope for bias. Analyses of alcohol brief intervention focused on adults drinking at risky levels who reported having visited their GP surgery in the past year but did not take into account the number of GP surgery visits over this period. If there were differences in frequency of visits by exposure variables (e.g., smoking status), this may have led to differences in opportunities to receive brief intervention, potentially biasing the results. Replication in another sample with adjustment for frequency of GP surgery visits would be useful to confirm our results.

We analysed use of different categories of non-combustible nicotine products as a single variable, but associations may differ across the product categories. For example, NRT is a licensed smoking cessation treatment (though mostly bought privately over the counter) and primarily used as a cessation aid while e-cigarettes, heated tobacco products, and nicotine pouches are commercial products that are often used outside of attempts to quit smoking^31^.

Our data collection focused on housed people, so our results do not capture certain sub-populations (e.g., people experiencing homelessness, those in residential treatment for other substance dependencies, those in prison)^32,33^. In addition, the cross-sectional nature of the survey prevented us from examining trajectories within individuals, which would be helpful when examining transitions and interactions between alcohol and smoking status and non-combustible nicotine use^34^. Further research is required to provide further insight on this.

Finally, it is possible that associations may have changed over time in the context of the Covid-19 pandemic, declining trends in smoking prevalence^19^ and increases in non-combustible nicotine use (specifically e-cigarettes^35^), and age-related differences in trends in non-combustible nicotine use^35^ and alcohol consumption^36^. Given the large number of (pre-registered) cross-sectional analyses, we did not explore temporal changes in associations, but this would be an interesting direction for future research.

## Conclusions

Smoking – and to a lesser extent, use of non-combustible nicotine–is associated with higher levels of alcohol consumption and risky drinking. Among adults engaging in risky drinking, those who smoke are more likely than those who do not to report receiving alcohol brief intervention in primary care. Those who attempt to quit smoking are more likely to report trying to reduce their alcohol consumption than those who do not.

### Methods

#### Pre-registration

The study protocol, research questions, and analysis plan were pre-registered on Open Science Framework (https://osf.io/3fau6/). We made one amendment, which was to include smoking status as an additional covariate in adjusted models testing associations between non-combustible nicotine use and each outcome and vice versa. We report results without this adjustment (per our pre-registered analysis plan) in the table footnotes. Following peer review, we repeated our analyses of associations with alcohol consumption excluding participants who reported not drinking at all (AUDIT-C = 0) from the sample.

### Design

Data were drawn from the Smoking and Alcohol Toolkit Study, an ongoing monthly cross-sectional survey of a nationally-representative representative sample of adults in England^37–39^. The study uses a hybrid of random probability and simple quota sampling to select a new sample of approximately 1,700 adults each month.

Full details of the sampling procedure are provided elsewhere^37,39^. Briefly, England is split into 165,665 output areas, each comprising approximately 300 households. These areas are then stratified according to established geo-demographic characteristics and geographic region, then randomly selected into an interviewer’s list. Interviews are held with one household member until quotas based on factors influencing the probability of being at home (i.e., working status, age and gender) are fulfilled. This hybrid form of random probability and quota sampling is considered superior to conventional quota sampling. Here, the choice of households to approach is limited by the random allocation of small output areas and rather than being allocated specific households in advance, interviewers can choose which households within these small geographic areas are most likely to fulfil their quotas. Therefore, unlike random probability sampling, it is not appropriate to record the response rate.

Data were collected monthly through face-to-face computer-assisted interviews up to February 2020. However, social distancing restrictions under the Covid-19 pandemic meant that no data were collected in March 2020, and data from April 2020 onwards have been collected via telephone. The telephone-based data collection relies upon a similar combination of random location and quota sampling, and weighting approach as the face-to-face interviews and comparisons of the two data collection modalities indicate good comparability^40–42^.

For the present study, we used data collected from respondents to the survey in the period from March 2014 (the first wave to collect detailed information on alcohol use) to December 2023 (the most recent data available at the time of analysis). We excluded those aged under 18, who cannot legally be sold alcohol or tobacco in England.

### Measures

#### Outcomes

Alcohol consumption was assessed with the three-item AUDIT-C^43^, a screening tool developed by the World Health Organization. Questions are framed in the context of drinking behaviour over the last 6 months. We analysed: (i) mean AUDIT-C scores (range: 0–12, with higher scores indicating higher levels of consumption); and (ii) the proportion drinking at increasing and higher risk (‘risky’) levels (defined as an AUDIT-C score ≥ 5). Participants who did not drink at all were included in the sample (and scored 0 on the AUDIT-C).

Receipt of alcohol brief intervention was assessed among participants drinking at risky levels with the question: ‘In the last 12 months, has a doctor or other health worker within your GP surgery discussed your drinking?

1. Yes, a doctor or other health worker within my GP surgery asked about my drinking;
2. Yes, a doctor or other health worker within my GP surgery offered advice about cutting down on my drinking;
3. Yes, a doctor or other health worker within my GP surgery offered help or support within the surgery to help me cut down;
4. Yes, a doctor or other health worker within my GP surgery referred me to an alcohol service or advised me to seek specialist help;
5. No, I have seen a doctor or health worker within my GP surgery in the last 12 months but did not discuss my drinking;
6. No, I have not seen a doctor or health worker within my GP surgery in last 12 months.’

Those who responded ‘yes’ were able to select multiple responses between 1 and 4 to indicate all types of advice they received. Those who responded ‘no’ were able to select only one response option (5 or 6). Receipt of brief intervention was coded 1 for those who selected response options 2, 3, or 4 and 0 for those who selected response options 1 or 5. Those who responded that they had not seen a doctor or health worker within their GP surgery in the last year (response option 6) were excluded from analyses of this outcome.

Alcohol reduction attempts were assessed among participants who engaged in risky drinking with the question: ‘How many attempts to restrict your alcohol consumption have you made in the last 12 months (e.g. by drinking less, choosing lower strength alcohol or using smaller glasses)? Please include all attempts you have made in the last 12 months, whether or not they were successful, and any attempt that you are currently making.’ Those who reported making at least one attempt to cut down drinking in the past year were coded 1, else they were coded 0.

#### Exposures

Smoking status was assessed by asking participants which of the following best applies to them:

- ‘I smoke cigarettes (including hand-rolled) every day’.
- ‘I smoke cigarettes (including hand-rolled), but not every day’.
- ‘I do not smoke cigarettes at all, but I do smoke tobacco of some kind (e.g. pipe, cigar or shisha)’.
- ‘I have stopped smoking completely in the last year’.
- ‘I stopped smoking completely more than a year ago’.
- ‘I have never been a smoker (i.e. smoked for a year or more)’.

Responses *a* to *c* were considered current smoking, *d* or *e* former smoking, and *f* never regular smoking. Those who responded *a* to *d* were considered to have smoked in the past year (the group of interest for analyses of attempts to quit smoking).

Use of non-combustible nicotine was assessed within several questions that ask participants about use of a range of nicotine products. Participants who currently smoked were asked ‘Do you regularly use any of the following in situations when you are not allowed to smoke?’; those who had smoked in the past year were asked ‘Can I check, are you using any of the following either to help you stop smoking, to help you cut down or for any other reason at all?’; and those who had not smoked in the past year were asked ‘Can I check, are you using any of the following?’. Those who reported using an e-cigarette, NRT (nicotine gum, lozenges/tablets, inhaler, nasal spray, patch, or mouth spray), heated tobacco products (‘heat-not-burn cigarette (e.g., iQOS, heatsticks)’), or nicotine pouches (‘tobacco-free nicotine pouch/pod or ‘white pouches’ that you place on your gum’) were considered to use non-combustible nicotine (coded 1), else they were coded 0.

Level of tobacco dependence was assessed among participants who currently smoked by asking them to self-report ratings of the strength of urges to smoke over the past 24 h [not at all (coded 0), slight (1), moderate (2), strong (3), very strong (4) and extremely strong (5)]. This variable was also coded ‘0’ for participants who responded ‘not at all’ to the (separate) question: ‘How much of the time have you spent with the urge to smoke?’^44^. This measure has been validated and performs at least as well as the Fagerström Test of Cigarette Dependence and the Heaviness of Smoking Index in predicting the outcome of cessation while not being subject to bias due to population-level changes in cigarette consumption over the time period of the study^44^.

Attempts to quit smoking were assessed among participants who smoked in the past year with the question ‘How many serious attempts to stop smoking have you made in the last 12 months? By serious attempt I mean you decided that you would try to make sure you never smoked again. Please include any attempt that you are currently making and please include any successful attempt made within the last year’. Those who reported making at least one serious quit attempt in the past year were coded 1, else they were coded 0.

#### Covariates

Sociodemographic characteristics included age, gender, occupational social grade, and region. Age was categorised as 18–24, 25–34, 35–44, 45–54, 55–64, and ≥ 65 years. Gender was self-reported as man, women, or other; those identifying as non-binary were excluded from analyses adjusting for gender due to low numbers. Occupational social grade was categorised as ABC1 (which includes managerial, professional and intermediate occupations) vs. C2DE (which includes small employers and own-account workers, lower supervisory and technical occupations, and semi‐routine and routine occupations, never workers and long‐term unemployed). This occupational measure of social grade is a valid index of SES, widely used in research in UK populations, which is particularly relevant in the context of tobacco use^45^ and alcohol consumption^46^. Region in England was categorised as North, Midlands, or South.

Survey year was modelled using restricted cubic splines, with three knots placed at equal quantiles of the data to account for non-linear trends.

### Statistical analysis

Data were analysed in R version 4.2.1. We excluded participants with missing data on alcohol consumption or smoking status. Missing cases on other variables were excluded on a per-analysis basis (see Table S1 for details). The Smoking and Alcohol Toolkit Study uses raking to weight the sample to match the population in England^38^. The following analyses used weighted data.

Alcohol consumption (AUDIT-C) was assessed in each wave, but other alcohol outcomes were assessed less frequently (e.g., in alternate months) according to availability of competitive research funding so samples for these analyses were smaller.

We examined the following associations (see the protocol for further information: https://osf.io/3fau6/):

- Among adults: (a) mean AUDIT-C scores and (b) the proportion reporting risky drinking, by smoking status and use of non-combustible nicotine.
- Among adults who currently smoked: (a) mean AUDIT-C scores and (b) the proportion reporting risky drinking, by level of tobacco dependence.
- Among adults who engaged in risky drinking and visited their GP surgery in the past year: (a) receipt of alcohol brief intervention and (b) making ≥ 1 past-year alcohol reduction attempt, by smoking status and use of non-combustible nicotine.
- Among adults who engaged in risky drinking and currently smoked: making ≥ 1 past-year alcohol reduction attempt, by level of tobacco dependence.
- Among adults who engaged in risky drinking and smoked in the past year: making ≥ 1 past-year alcohol reduction attempt, by whether they had made ≥ 1 past-year attempt to quit smoking.

For each of these, we reported descriptive data (mean or % with 95% confidence intervals [CIs]) on the outcome stratified by the exposure and used regression models (linear or logistic, as appropriate) to test associations of the exposure with the outcome, with and without adjustment for covariates. All adjusted models controlled for sociodemographic characteristics and survey year.

Participants who dually smoked cigarettes and used non-combustible nicotine were included in the analysis. Models with smoking status as the exposure were additionally adjusted for use of non-combustible nicotine, and vice versa, to offer insight into independent associations. Smoking status and non-combustible nicotine use are closely related, and plausible confounders in this context. Models with receipt of alcohol brief intervention and alcohol reduction attempts as the outcome were additionally adjusted for level of alcohol consumption (AUDIT-C score).

## Electronic supplementary material

Below is the link to the electronic supplementary material.

Supplementary Material 1

## Data Availability

The data used in these analyses are available on Open Science Framework (https://osf.io/3fau6/).

## References

[1] Griswold, M. G. et al. Alcohol use and burden for 195 countries and territories, 1990–2016: A systematic analysis for the global burden of Disease Study 2016. *Lancet* **392**, 1015–1035 (2018). 30146330 10.1016/S0140-6736(18)31310-2 PMC6148333

[2] Shield, K. et al. National, regional, and global burdens of disease from 2000 to 2016 attributable to alcohol use: A comparative risk assessment study. *Lancet Public Health*. **5**, e51–e61 (2020). 31910980 10.1016/S2468-2667(19)30231-2

[3] Bush, K. et al. The AUDIT alcohol consumption questions (AUDIT-C): An effective brief screening test for problem drinking. *Arch. Intern. Med.* **158**, 1789–1795 (1998). 9738608 10.1001/archinte.158.16.1789

[4] Buss, V., Brown, J. & Kock, L. *Latest Trends on Alcohol Consumption in England from the Alcohol Toolkit Study*. (2025). https://www.alcoholinengland.info/graphs/monthly-tracking-kpi

[5] US Department of Health and Human Services. Eighth Special Report to the US Congress on Alcohol and Health. (1993).

[6] National Institute for Health and Care Excellence. *Alcohol-Use Disorders: Prevention*. (2010). https://www.nice.org.uk/guidance/ph24

[7] Burton, R. et al. The independent and joint risks of alcohol consumption, smoking, and excess weight on morbidity and mortality: A systematic review and meta-analysis exploring synergistic associations. *Public Health*. **226**, 39–52 (2024). 38000113 10.1016/j.puhe.2023.10.035

[8] Talamini, R. et al. Combined effect of tobacco and alcohol on laryngeal cancer risk: A case-control study. *Cancer Causes Control CCC*. **13**, 957–964 (2002). 12588092 10.1023/a:1021944123914

[9] Pelucchi, C., Gallus, S., Garavello, W. & Bosetti, C. La Vecchia, C. Cancer risk associated with alcohol and tobacco use: Focus on upper aero-digestive tract and liver. *Alcohol Res. Health*. **29**, 193–198 (2006). 17373408 PMC6527045

[10] Le Strat, Y., Ramoz, N. & Gorwood, P. In alcohol-dependent drinkers, what does the presence of nicotine dependence tell us about psychiatric and addictive disorders comorbidity? *Alcohol Alcohol Oxf. Oxfs.* **45**, 167–172 (2010). 10.1093/alcalc/agp094 20089545

[11] Benowitz, N. L., Jones, R. T. & Jacob, P. Additive cardiovascular effects of nicotine and ethanol. *Clin. Pharmacol. Ther.* **40**, 420–424 (1986). 3757405 10.1038/clpt.1986.200

[12] Garnett, C., Oldham, M., Brose, L., Cheeseman, H. & Cox, S. Prevalence and characteristics of co-occurrence of smoking and increasing-and-higher-risk drinking: A population survey in England. *Addict. Behav.* **150**, 107928 (2024). 38091779 10.1016/j.addbeh.2023.107928

[13] Beard, E., West, R., Michie, S. & Brown, J. Association between smoking and alcohol-related behaviours: A time–series analysis of population trends in England. *Addict. Abingdon Engl.* **112**, 1832–1841 (2017). 10.1111/add.13887 PMC5600127 28556467

[14] Garnett, C., Oldham, M., Shahab, L., Tattan-Birch, H. & Cox, S. Characterising smoking and smoking cessation attempts by risk of alcohol dependence: A representative, cross-sectional study of adults in England between 2014–2021. *Lancet Reg. Health Eur.* **18**, (2022). 10.1016/j.lanepe.2022.100418 PMC9257647 35814338

[15] Kahler, C. W., Spillane, N. S. & Metrik, J. Alcohol use and initial smoking lapses among heavy drinkers in smoking cessation treatment. *Nicotine Tob. Res.* **12**, 781–785 (2010). 20507898 10.1093/ntr/ntq083 PMC2893295

[16] Room, R., Babor, T. & Rehm, J. Alcohol and public health. *Lancet* **365**, 519–530 (2005). 15705462 10.1016/S0140-6736(05)17870-2

[17] Fagerström, K. The epidemiology of smoking. *Drugs* **62**, 1–9 (2002). 12109931 10.2165/00003495-200262002-00001

[18] Frie, J. A., Nolan, C. J., Murray, J. E. & Khokhar, J. Y. Addiction-related outcomes of nicotine and alcohol co-use: New insights following the rise in vaping. *Nicotine Tob. Res.* **24**, 1141–1149 (2022). 34758090 10.1093/ntr/ntab231 PMC9278825

[19] Office for National Statistics. *Adult Smoking Habits in the UK: 2022*. (2023). https://www.ons.gov.uk/peoplepopulationandcommunity/healthandsocialcare/healthandlifeexpectancies/bulletins/adultsmokinghabitsingreatbritain/2022

[20] Funk, D., Marinelli, P. W. & Lê, A. D. Biological processes underlying co-use of alcohol and nicotine: Neuronal mechanisms, crosstolerance, and genetic factors. *Alcohol Res. Health*. **29**, 186–192 (2006). 17373407 PMC6527043

[21] Perkins, K. A. Combined effects of nicotine and alcohol on subjective, behavioral and physiological responses in humans. *Addict. Biol.* **2**, 255–268 (1997). 26735782 10.1080/13556219772552

[22] Tyndale, R. F. Genetics of alcohol and tobacco use in humans. *Ann. Med.* **35**, 94–121 (2003). 12795339 10.1080/07853890310010014

[23] McNeill, A. et al. *Nicotine Vaping in England: An Evidence Update Including Health Risks and Perceptions, September 2022. A Report Commissioned by the Office for Health Improvement and Disparities*. (2022). https://www.gov.uk/government/publications/nicotine-vaping-in-england-2022-evidence-update

[24] Brown, J. et al. Comparison of brief interventions in primary care on smoking and excessive alcohol consumption: A population survey in England. *Br. J. Gen. Pract.* **66**, e1–e9 (2016). 26719481 10.3399/bjgp16X683149 PMC4684029

[25] Buss, V. H. et al. Alcohol and smoking brief interventions by socioeconomic position: A population-based, cross-sectional study in Great Britain. *BJGP Open.* **7**, (2023). BJGPO.2023.0087. 10.3399/BJGPO.2023.0087 PMC11176676 37549977

[26] Aira, M., Kauhanen, J., Larivaara, P. & Rautio, P. Differences in brief interventions on excessive drinking and smoking by primary care physicians: Qualitative study. *Prev. Med.* **38**, 473–478 (2004). 15020181 10.1016/j.ypmed.2003.11.023

[27] Buss, V., West, R., Kock, L., Kale, D. & Brown, J. Monthly trends on smoking in England from the Smoking Toolkit Study. (2024). https://smokinginengland.info/graphs/monthly-tracking-kpi

[28] Jiang, N. & Ling, P. M. Reinforcement of smoking and drinking: Tobacco marketing strategies linked with alcohol in the United States. *Am. J. Public. Health*. **101**, 1942–1954 (2011). 21852637 10.2105/AJPH.2011.300157 PMC3215402

[29] Royal College of Physicians. *E-Cigarettes and Harm Reduction: An Evidence Review*. (2024). https://www.rcp.ac.uk/policy-and-campaigns/policy-documents/e-cigarettes-and-harm-reduction-an-evidence-review/

[30] Brown, J. et al. Are recent attempts to quit smoking associated with reduced drinking in England? A cross-sectional population survey. *BMC Public. Health*. **16**, 535 (2016). 27443348 10.1186/s12889-016-3223-6 PMC4957412

[31] Jackson, S. E., Beard, E. & Brown, J. Smokers’ use of E-cigarettes in situations where smoking is not permitted in England: Quarterly trends 2011–2020 and associations with sociodemographic and smoking characteristics. *Nicotine Tob. Res.* **23**, 1831–1838 (2021). 34089607 10.1093/ntr/ntab119 PMC8496468

[32] Rehm, J., Kilian, C. & Manthey, J. Future of surveys in the alcohol field. *Drug Alcohol Rev.* **40**, 176–178 (2021). 32959438 10.1111/dar.13180

[33] J, R., Kd, C. K. P. R. & J, M. S. The elusiveness of representativeness in general population surveys for alcohol. *Drug Alcohol Rev.* **40**, (2021). 10.1111/dar.13148 32830351

[34] Pacek, L. R. et al. Current tobacco use, nicotine dependence, and transitions across stages of alcohol involvement: A latent transition analysis approach. *Int. J. Methods Psychiatr Res.* **28**, e1789 (2019). 31141253 10.1002/mpr.1789 PMC6791727

[35] Tattan-Birch, H., Brown, J., Shahab, L., Beard, E. & Jackson, S. E. Trends in vaping and smoking following the rise of disposable e-cigarettes: a repeat cross-sectional study in England between 2016 and 2023. *Lancet Reg. Health Eur.* 100924. 10.1016/j.lanepe.2024.100924 (2024). 10.1016/j.lanepe.2024.100924 PMC11281926 39070753

[36] Oldham, M. et al. The decline in youth drinking in England—is everyone drinking less? A quantile regression analysis. *Addiction* **115**, 230–238 (2020). 31560404 10.1111/add.14824 PMC7004203

[37] Beard, E. et al. Protocol for a national monthly survey of alcohol use in England with 6-month follow-up: ‘The Alcohol Toolkit Study’. *BMC Public. Health*. **15**, 230 (2015). 25884652 10.1186/s12889-015-1542-7 PMC4363185

[38] Fidler, J. A. et al. The smoking toolkit study’: A national study of smoking and smoking cessation in England. *BMC Public. Health*. **11**, 479 (2011). 21682915 10.1186/1471-2458-11-479 PMC3145589

[39] Kock, L. et al. Protocol for expansion of an existing national monthly survey of smoking behaviour and alcohol use in England to Scotland and Wales: The smoking and alcohol toolkit study. *Wellcome Open. Res.* **6**, 67 (2021). 34458587 10.12688/wellcomeopenres.16700.1 PMC8370132

[40] Jackson, S. E., Beard, E., Angus, C., Field, M. & Brown, J. Moderators of changes in smoking, drinking and quitting behaviour associated with the first COVID-19 lockdown in England. *Addiction* **117**, 772–783 (2022). 34431577 10.1111/add.15656 PMC8652848

[41] Jackson, S. E., Garnett, C., Shahab, L., Oldham, M. & Brown, J. Association of the Covid-19 lockdown with smoking, drinking, and attempts to quit in England: An analysis of 2019–2020 data. *Addiction* **116**, 1233–1244 (2021). 33089562 10.1111/add.15295 PMC8436745

[42] Kock, L., Tattan-Birch, H., Jackson, S., Shahab, L. & Brown, J. Socio-demographic, smoking and drinking characteristics in GB: A comparison of independent telephone and face-to-face smoking and Alcohol Toolkit surveys conducted in March 2022. *Qeios*10.32388/CLXK4D (2022).

[43] Babor, T. F., Higgins-Biddle, J. C., Saunders, J. B., Monteiro, M. G. & Organization, W. H. AUDIT: The alcohol use disorders identification test: Guidelines for use in primary health care. (2001).

[44] Fidler, J. A., Shahab, L. & West, R. Strength of urges to smoke as a measure of severity of cigarette dependence: Comparison with the Fagerström test for nicotine dependence and its components. *Addict. Abingdon Engl.* **106**, 631–638 (2011). 10.1111/j.1360-0443.2010.03226.x 21134020

[45] Kotz, D. & West, R. Explaining the social gradient in smoking cessation: It’s not in the trying, but in the succeeding. *Tob. Control*. **18**, 43–46 (2009). 18936053 10.1136/tc.2008.025981

[46] Beard, E. et al. Associations between socio-economic factors and alcohol consumption: A population survey of adults in England. *PLOS ONE*. **14**, e0209442 (2019). 30716098 10.1371/journal.pone.0209442 PMC6361426

