# Supplementary material for: Smoking, and to a lesser extent non-combustible nicotine use, is associated with higher levels of alcohol consumption and risky drinking
Source: Sci Rep. 2025 Feb 26;15:6851. doi: 10.1038/s41598-025-89750-2 (PMC11865552; doi:10.1038/s41598-025-89750-2)
Supplement: Supplementary file 1 — Supplementary Material 1 [file 41598_2025_89750_MOESM1_ESM.pdf]

Table S1. Weighted sample characteristics

	%[1]
**All adults (n=188,787)**	
Age	
Mean (SD)	48.0 (18.6)
18-24	12.3
25-34	17.1
35-44	16.4
45-54	17.3
55-64	14.7
≥65	22.2
Gender	
Men	48.9
Women	50.9
Other	0.3
Missing	147
Occupational social grade	
ABC1 (more advantaged)	55.6
C2DE (less advantaged)	44.4
Region	
North	28.1
Midlands	30.3
South	41.7
AUDIT-C score, mean (SD)	3.09 (2.90)
Increasing and higher risk drinking	
No	71.0
Yes	29.0
Smoking status	
Never	61.7
Former	21.0
Current	17.3
Use of non-combustible nicotine	
No	91.2
Yes	8.8
**Adults who engaged in risky drinking (n=53,710)**	
≥1 past-year alcohol reduction attempt	
No	71.2
Yes	28.8
Missing	5,480

Table is continued on the next page.

Table S1. *continued*

	%[1]
**Adults who engaged in risky drinking and visited their GP in the past 12 months (n=31,815)**	
Receipt of alcohol brief intervention	
No	97.1
Yes	2.9
**Adults who currently smoked (n=31,730)**	
Strength of urges to smoke	
Not at all	14.8
Slight	18.4
Moderate	43.5
Strong	16.1
Very strong	5.0
Extremely strong	2.3
Missing	568
**Adults who smoked in the past year (n=34,795)**	
≥1 past-year attempt to quit smoking	
No	66.1
Yes	33.9
Missing	1,277

[1] Data are shown as weighted percentages, unless otherwise specified. Sample sizes (including numbers of missing cases) are unweighted.

Table S2. Alcohol consumption among adults in England who drink alcohol (AUDIT-C ≥1), by smoking status, use of non-combustible nicotine, and (among those who currently smoked) strength of urges to smoke

		AUDIT-C score			Risky drinking (AUDIT-C ≥5)		
	N[1]	Mean [95%CI]	*B* [95%CI]	B_{adj} [95%CI][2]	% [95%CI]	OR [95%CI]	OR_{adj} [95%CI][2]
**All adults**							
Smoking status							
Never	79,436	3.95 [3.93–3.97]	Ref	Ref	34.4 [34.1–34.8]	Ref	Ref
Former	32,522	4.61 [4.58–4.64]	0.66 [0.62; 0.69]	0.72 [0.68; 0.75]	45.3 [44.7–45.9]	1.58 [1.53; 1.62]	1.80 [1.75; 1.86]
Current	21,877	5.23 [5.19–5.27]	1.28 [1.24; 1.33]	1.11 [1.07; 1.16]	55.4 [54.7–56.1]	2.36 [2.29; 2.44]	2.22 [2.14; 2.30]
Use of non-combustible nicotine							
No	122,434	4.23 [4.22–4.25]	Ref	Ref	39.1 [38.8–39.3]	Ref	Ref
Yes	11,401	5.24 [5.18–5.29]	1.00 [0.95; 1.06]	0.24 [0.18; 0.30]	55.6 [54.7–56.6]	1.96 [1.88; 2.04]	1.15 [1.09; 1.21]
**Adults who currently smoked**							
Strength of urges to smoke							
Not at all	3,439	5.25 [5.15–5.36]	Ref	Ref	56.9 [55.1–58.7]	Ref	Ref
Slight	3,979	5.16 [5.07–5.26]	-0.05 [-0.17; 0.07]	0.03 [-0.09; 0.15]	55.7 [54.0–57.3]	0.97 [0.89; 1.06]	1.03 [0.94; 1.13]
Moderate	9,187	5.10 [5.04–5.17]	-0.11 [-0.22; -0.01]	0.08 [-0.02; 0.18]	53.6 [52.5–54.7]	0.90 [0.84; 0.97]	1.04 [0.96; 1.12]
Strong	3,428	5.33 [5.22–5.43]	0.09 [-0.04; 0.23]	0.30 [0.17; 0.43]	55.9 [54.1–57.7]	0.98 [0.89; 1.08]	1.15 [1.04; 1.28]
Very strong	1,001	5.70 [5.49–5.92]	0.47 [0.24; 0.70]	0.66 [0.44; 0.88]	58.2 [54.9–61.5]	1.06 [0.91; 1.23]	1.24 [1.06; 1.45]
Extremely strong	475	6.61 [6.29–6.93]	1.34 [1.01; 1.67]	1.46 [1.13; 1.78]	70.0 [65.6–74.3]	1.74 [1.41; 2.15]	2.10 [1.68; 2.63]

CI, confidence interval. OR, odds ratio.

[1] Unweighted sample size.

[2] All models were adjusted for age, gender, occupational social grade, and survey year (modelled non-linearly using restricted cubic splines, three knots). Models with smoking status as the exposure were also adjusted for use of non-combustible nicotine. Models with use of non-combustible nicotine as the exposure were also adjusted for smoking status.

Table S3. Receipt of different types of advice on alcohol from a health professional within their GP surgery among adults in England who engaged in risky drinking and visited their GP, by smoking status and use of non-combustible nicotine

		**Advice received, % [95%CI]**				
	***N*[1]**	**Asked about my drinking**	**Offered advice about cutting down on my drinking**	**Offered help or support within the surgery to help me cut down**	**Referred me to an alcohol service or advised me to seek specialist help**	**Did not discuss my drinking**
**Adults who engaged in risky drinking and visited their GP**						
Smoking status						
Never	15,776	11.1 [10.6–11.6]	1.93 [1.70–2.16]	0.53 [0.40–0.65]	0.37 [0.26–0.47]	86.9 [86.3–87.4]
Former	9,382	13.9 [13.2–14.7]	3.50 [3.09–3.91]	0.73 [0.53–0.92]	0.58 [0.41–0.75]	83.2 [82.4–84.0]
Current	6,657	11.5 [10.7–12.3]	3.75 [3.28–4.23]	1.47 [1.17–1.76]	2.26 [1.87–2.65]	83.8 [82.8–84.7]
Use of non-combustible nicotine						
No	28,276	11.9 [11.5–12.3]	2.58 [2.38–2.77]	0.68 [0.57–0.78]	0.73 [0.62–0.84]	85.4 [84.9–85.8]
Yes	3,539	12.5 [11.3–13.7]	4.25 [3.53–4.98]	1.63 [1.18–2.07]	1.65 [1.19–2.11]	83.3 [81.9–84.6]

CI, confidence interval.
[1] Unweighted sample size.
